# Dual molecular and clinical machine-learning prognostic modeling in pancreatic ductal adenocarcinoma: a chaperone-mediated autophagy–based framework integrating multi-cohort molecular signatures and a single-center clinical nomogram

**DOI:** 10.3389/fcell.2026.1939520

**Published:** 2026-09-09

**Authors:** Qingyan Kou, Shengxian Qiao, Zhenyuan Liu, Zhichao Wu, Wenbin Zhao, Xu Zhang

**Affiliations:** General Surgery Department, Qingdao Central Hospital, University of Health and Rehabilitation Sciences, Qingdao, China

**Keywords:** chaperone-mediated autophagy, clinical nomogram, KRT19, machine learning, pancreatic ductal adenocarcinoma, prognostic model, tumor immune microenvironment

## Abstract

**Background:**

Pancreatic ductal adenocarcinoma (PDAC) is characterized by marked molecular, cellular, and clinical heterogeneity. Chaperone-mediated autophagy (CMA) supports adaptation to metabolic and environmental stress, but its cell type-specific distribution and prognostic relevance in PDAC remain unclear.

**Methods:**

Single-cell RNA sequencing data from GSE212966 were analyzed to characterize CMA-related transcriptional states in PDAC and adjacent non-tumor tissues. Bulk transcriptomic data from TCGA-PAAD were used for differential expression analysis, weighted gene co-expression network analysis, and molecular model development, while ICGC PACA-CA and PACA-AU served as independent validation cohorts. Multiple survival machine-learning approaches were compared to establish a CMA-related prognostic model. Hallmark pathway activity, immune infiltration, and predicted drug sensitivity were evaluated between risk groups. KRT19, the highest-weighted model gene, was selected for *in vitro* validation. In parallel, an independent single-center cohort of 468 patients was analyzed using eight survival machine-learning methods to identify clinical prognostic factors and construct a nomogram.

**Results:**

CMA-related transcriptional activity varied among cell types, with macrophages showing prominent scores and PDAC-derived macrophages exhibiting higher CMA scores than those from adjacent tissues. Integration of TCGA differential expression analysis and WGCNA identified 105 candidate genes. The StepCox [forward] plus random survival forest model showed favorable overall performance, with C-index values of 0.903, 0.678, and 0.733 in the TCGA, PACA-CA, and PACA-AU cohorts, respectively. High molecular risk was associated with enhanced glycolytic, proliferative, and cell cycle-related signaling, increased M0 macrophages, reduced CD8^+^ T cells, and differential predicted drug sensitivity. KRT19 overexpression promoted PDAC cell proliferation, colony formation, migration, and invasion. In the single-center cohort, N stage, CA125, vascular tumor thrombus, and total bilirubin ranked highest in weighted prognostic importance. The clinical nomogram achieved AUC values of 0.661 and 0.750 for 1- and 3-year overall survival, respectively.

**Conclusion:**

This study identified CMA-related cellular heterogeneity, established a molecular prognostic model that retained prognostic discrimination in two independent validation cohorts, demonstrated the functional relevance of KRT19, and developed an independent clinical prediction tool. These molecular and clinical models provide complementary perspectives on PDAC prognosis and warrant further evaluation in matched prospective cohorts.

## 1 Introduction

Pancreatic ductal adenocarcinoma (PDAC) is one of the most aggressive and lethal malignancies of the digestive system. It is characterized by insidious early symptoms, frequent diagnosis at locally advanced or metastatic stages, limited therapeutic response, and poor long-term survival (Kleeff et al., 2016). Despite ongoing refinements in surgical technique, cytotoxic chemotherapy regimens, molecularly targeted agents, and immune-based approaches, survival gains for PDAC patients have remained modest (Halbrook et al., 2023). The highly malignant biological behavior of PDAC is closely associated with KRAS-driven molecular alterations, prominent desmoplastic stroma, poor vascular perfusion, hypoxia and nutrient limitation, metabolic reprogramming, and an immunosuppressive tumor microenvironment (Sherman and Beatty, 2023; Tao et al., 2021; Olaoba et al., 2024). Although tumor-node-metastasis (TNM) staging and conventional clinicopathological indicators provide important information regarding disease extent and patient prognosis, they remain insufficient to fully explain the marked outcome heterogeneity among patients with apparently similar clinical presentations. Molecular features may capture biological processes that are not reflected by routine clinical assessment, whereas machine-learning integration of readily available clinicopathological variables may improve the prognostic use of existing clinical information. Therefore, complementary evaluation of molecular and clinical prognostic factors may provide a more comprehensive basis for patient risk stratification and individualized management.

Autophagy is an important mechanism by which tumor cells adapt to harsh microenvironmental conditions. It plays essential roles in maintaining cellular homeostasis, removing damaged organelles, regulating the supply of metabolic substrates, and responding to therapeutic stress (Amaravadi et al., 2019). Unlike macroautophagy, chaperone-mediated autophagy (CMA) is a selective lysosomal degradation process. It generally depends on heat shock protein family A member 8 (HSPA8/HSC70) to recognize KFERQ-like motifs in substrate proteins and on lysosome-associated membrane protein type 2A (LAMP2A) to mediate substrate translocation into lysosomes for degradation (Kaushik and Cuervo, 2018). Previous studies have suggested that CMA is involved not only in protein quality control and metabolic regulation, but also in tumor cell survival, stress adaptation, therapeutic resistance, and immune microenvironment remodeling (Liu et al., 2023; Kon et al., 2011). PDAC tissues are commonly exposed to hypoxia, nutrient deprivation, and inflammatory stimulation, and both tumor cells and surrounding immune and stromal cells may rely on autophagy- and lysosome-related programs to maintain their functional states (Yang et al., 2018). However, how CMA-linked gene expression programs are distributed among the diverse cell types within the PDAC microenvironment, and how these programs relate to patient prognosis and immune composition, has not been thoroughly investigated.

The advent of single-cell RNA sequencing (scRNA-seq) has made it possible to dissect tumor tissue at a resolution that bulk profiling cannot achieve, revealing which cell populations are actually responsible for a given transcriptional signal rather than lumping tumor, immune, and stromal contributions together indiscriminately (Peng et al., 2019). At the same time, large-scale repositories such as The Cancer Genome Atlas (TCGA) and International Cancer Genome Consortium (ICGC) contribute the sample depth and long-term follow-up data necessary for developing and evaluating clinically meaningful survival models (Chang et al., 2013; Hudson et al., 2010). Within such cohorts, WGCNA offers a way to uncover clusters of co-regulated genes tied to a trait of interest, while survival-oriented machine-learning approaches can subsequently narrow down which of these genes carry genuine prognostic weight (Langfelder and Horvath, 2008; Wang and Li, 2017; Gui and Li, 2005). In parallel, routinely collected clinicopathological and laboratory variables remain the most immediately accessible sources of prognostic information in real-world clinical practice. Applying survival-oriented machine-learning approaches to an independent clinical cohort may help identify clinically accessible prognostic factors and provide a practical reference for interpreting transcriptome-derived risk stratification. Integrating cellular-resolution mapping, network-based molecular modeling, cross-cohort molecular validation, and independent clinical prognostic modeling may therefore provide complementary biological and clinically accessible perspectives on PDAC prognosis.

Based on this background, the present study established a complementary molecular and clinical framework to investigate prognostic heterogeneity in PDAC. The GSE212966 scRNA-seq dataset was first analyzed to characterize the cellular distribution of CMA-related transcriptional states. TCGA-PAAD and two independent ICGC cohorts were then used to identify CMA-related candidate genes and construct and validate a machine-learning-based molecular prognostic model. Hallmark pathway activity, immune infiltration, and predicted drug sensitivity were further examined to characterize the biological and potential therapeutic features associated with molecular risk stratification. KRT19, the highest-ranked gene in the final molecular model, was subsequently selected for *in vitro* functional validation. In parallel, an independent single-center cohort of 468 patients was analyzed using multiple survival machine-learning approaches to identify clinically accessible prognostic factors and develop a clinicopathological nomogram. By examining transcriptome-derived molecular risk and routinely available clinical predictors in complementary cohorts, this study aimed to provide a broader framework for prognostic assessment and translational exploration in PDAC.

## 2 Materials and methods

### 2.1 Data sources and study cohorts

This study included public transcriptomic datasets and an independent single-center clinical cohort to investigate PDAC prognosis from complementary molecular and clinical perspectives. Single-cell transcriptomic data were obtained from the Gene Expression Omnibus database under accession number GSE212966. This dataset included PDAC tumor tissues and adjacent non-tumor tissues (ADJ) and was used to characterize the cellular distribution of CMA-related transcriptional states (Barrett et al., 2013).

Bulk RNA-seq expression profiles and corresponding clinical information were obtained from the TCGA-PAAD cohort, which served as the molecular training cohort for differential expression analysis, WGCNA, and prognostic model construction. The ICGC PACA-CA and ICGC PACA-AU cohorts were used as independent external validation cohorts for the transcriptome-derived molecular risk model. For bulk transcriptomic analyses, gene identifiers were standardized across cohorts, and only samples with complete expression profiles, OS time, and survival status were retained. Candidate genes shared by the TCGA-PAAD, ICGC PACA-CA, and ICGC PACA-AU cohorts were used for model construction and validation.

In addition, an independent single-center cohort comprising 468 patients with PDAC from the General Surgery Department of Qingdao Central Hospital, University of Health and Rehabilitation Sciences, was included for clinical prognostic modeling. Routinely available demographic characteristics, pathological features, serum tumor markers, laboratory indicators, and survival follow-up information were collected. This cohort was analyzed independently to assess the prognostic information provided by clinically accessible variables. Because paired transcriptomic profiles were unavailable, the single-center cohort was not used as an external validation cohort for the CMA-related molecular signature.

### 2.2 CMA-related gene set and CMA score calculation

CMA-related genes were classified into three predefined categories: effector genes, positive regulators, and negative regulators (Khawaja et al., 2025). The complete scoring set comprised 84 genes, including 26 effector genes, 43 positive regulators, and 15 negative regulators. These genes are involved in autophagy-related effector functions, substrate recognition, lysosomal regulation, and signaling processes associated with CMA regulation. Because several components of this gene set also participate in broader autophagy and lysosomal processes, the resulting score was interpreted as a CMA-related transcriptional score rather than a direct measurement of functional CMA flux. The complete list of the 84 genes, their classifications, and their roles in the scoring framework are provided in Supplementary Table S1.

In both scRNA-seq and bulk expression matrices, CMA scores were calculated using the predefined 84-gene set according to the following formula:

$$\text{CMA score}=\left(2\;\times \text{effector sum}+\text{positive regulator sum}-\text{ negative regulator sum}\right)\;/\;84$$

Here, effector sum, positive regulator sum, and negative regulator sum represent the summed expression levels of the corresponding gene categories in each cell or bulk sample, and 84 represents the total number of genes included in the predefined scoring set (Supplementary Table S1).

### 2.3 Bioinformatics analysis in single-cell RNA sequencing

Processing and visualization of the single-cell data relied on the Seurat framework (Satija et al., 2015). Dimensionality reduction was performed based on the normalized expression matrix, and t-distributed stochastic neighbor embedding (tSNE) was used to visualize cell population distributions. Clusters were then assigned to specific cell identities according to well-established lineage marker genes, including Acinar cells, Macrophages, Fibroblast, NKT cell, Cancer cell, Neutrophil, Pancreas α cell, Endothelial cell, CD8T cell, Plasma cell, B cell, Mast cell, Treg cell, and Smooth muscle cell.

Once computed, CMA scores for individual cells were appended to the metadata table, after which their spread across cell identities, tissue-of-origin, and disease state was examined through a combination of tSNE-overlaid feature maps, violin plots, and sample-resolved heatmaps. This distribution served as the basis for pinpointing which cell types displayed particularly pronounced CMA-related states. For macrophages, CMA scores were first averaged within each independent biological sample to obtain a sample-level mean CMA score. These sample-level values, rather than individual cells, were then compared between PDAC and ADJ tissues using the Wilcoxon rank-sum test. Differential expression analysis between PDAC-derived and ADJ-derived macrophages was subsequently performed.

### 2.4 Bioinformatics analysis in bulk datasets and WGCNA

Differential expression analysis was performed between PDAC tumor and normal samples within the TCGA-PAAD cohort using the limma package (Ritchie et al., 2015). Differentially expressed genes were subsequently integrated with genes from the CMA-associated WGCNA module to identify candidate genes for downstream prognostic modeling. A design matrix was first constructed, followed by linear model fitting and empirical Bayes moderation. Significantly differentially expressed genes were defined as those with an adjusted P value <0.05 and |logFC| > 1. Differential expression results were visualized using a volcano plot.

WGCNA was applied to the TCGA-PAAD expression data to identify co-expression modules associated with CMA status. Hierarchical clustering of samples was first performed to assess sample quality and identify potential outliers. Candidate soft-thresholding powers were evaluated according to scale-free topology fit and mean connectivity, and a soft-thresholding power of 8 was selected for network construction. An unsigned network with an unsigned topological overlap matrix (TOM) was used. The principal blockwiseModules parameters were set as follows: minimum module size = 50 genes, maxBlockSize = 22,000, deepSplit = 2, reassignThreshold = 0, mergeCutHeight = 0.15, numericLabels = TRUE, and pamRespectsDendro = FALSE. Module eigengenes were subsequently correlated with both the continuous CMA score and the categorical CMA group, and the module showing the strongest and statistically significant association with the CMA score was selected as the principal CMA-associated module for downstream analysis. As a final step in this stage, genes belonging to the CMA-associated module were cross-referenced against the differentially expressed gene list from the TCGA bulk analysis, and the overlapping set was carried forward as candidate genes for the machine-learning modeling described in subsequent sections.

### 2.5 Machine-learning prognostic model

Using candidate genes as input features, we built a prognostic model in the TCGA-PAAD cohort after restricting the feature space to genes measured in common across TCGA, ICGC PACA-CA, and ICGC PACA-AU, retaining only samples with complete survival and expression data and standardizing expression values to minimize cross-cohort scale differences.

Eleven survival algorithms—RSF, StepCox (with forward, backward, and bidirectional selection), CoxBoost, Lasso, Ridge, Elastic Net, GBM, plsRcox, SuperPC, and survival-SVM—were evaluated both individually and in combination, with dimensionality-reduction methods (StepCox/CoxBoost/Lasso/Elastic Net) paired against downstream learners (RSF/GBM/SuperPC/survival-SVM) (Binder and Schumacher, 2008; Kourou et al., 2015; Van Belle et al., 2011; Bohannan et al., 2022). Every resulting model was trained exclusively on TCGA-PAAD and then applied without refitting to all three cohorts.

Predictive accuracy was gauged via the concordance index (C-index), computed in each cohort from the relationship between predicted risk and observed survival (Harrell et al., 1996). Rather than relying solely on training-set performance, we ranked candidate models by their mean C-index across TCGA, PACA-CA, and PACA-AU, selecting the combination that achieved the highest average while remaining robust in both external cohorts as the final model.

This selected model was then used to assign each patient a risk score, with median-based dichotomization separating subgroups in every cohort. Survival differences between the high-risk and low-risk groups were evaluated using Kaplan-Meier analysis and the log-rank test. Predictive accuracy at 1, 2, and 3 years was further assessed using time-dependent ROC analysis (Heagerty et al., 2000). These evaluations were performed in the training cohort and both external validation cohorts.

Finally, from the chosen RSF model we extracted variable importance rankings and retained the top 20 highest-weighted genes, which were subsequently examined via expression heatmaps and clinical/immune correlation analyses to characterize the model’s dominant molecular features.

### 2.6 Clinical association and cox regression analysis

We next asked how the CMA-derived risk score related to patients’ clinical characteristics by merging risk scores, risk group assignments, and a panel of clinicopathological variables within the TCGA-PAAD cohort. We began by examining whether the proportions of these clinical categories differed between risk strata, and then turned to the reverse question—whether risk scores themselves varied across T-stage subgroups (including a simplified T1–2 versus T3–4 comparison) and across clinical stage subgroups (including a Stage I–II versus Stage III–IV comparison).

Independence of the risk score as a prognostic determinant was tested through sequential Cox modeling: a univariate step first screened the risk score alongside individual clinical factors for their raw association with survival, after which a multivariate step entered the risk score together with the principal clinical covariates to see whether its predictive value held up once these factors were accounted for. Resulting hazard ratios, along with their 95% confidence intervals and corresponding P values, were compiled and rendered as forest plots for visual comparison.

### 2.7 Functional enrichment and Hallmark pathway analysis

Functional pathway activity was assessed using the Hallmark gene set collection drawn from MSigDB (Liberzon et al., 2015). Per-sample pathway enrichment scores were derived through GSVA (Hänzelmann et al., 2013), after which we contrasted these scores between the two risk strata, with the resulting differences presented as bar plots.

Beyond simple group comparisons, we examined the risk score’s linear association with each Hallmark pathway score and compiled these relationships into a correlation matrix. For pathways showing a clear link to survival outcomes, patients were split at the median pathway score into two activity tiers, and Kaplan-Meier curves were generated to visualize how pathway activity level tracked with overall survival.

### 2.8 Immune infiltration and drug sensitivity analysis

The tumor immune landscape was probed from three complementary angles—immune-related pathway activity, ssGSEA-based immune cell scoring, and CIBERSORT-based deconvolution—to see how each related to the CMA risk score. As a starting point, ssGSEA was applied to immune-associated gene sets to derive per-sample pathway scores, which were then compared across risk groups (Barbie et al., 2009). We then extended this approach to a panel of 28 immune cell subsets, scoring their relative infiltration via ssGSEA of subset-specific marker genes. Differences between risk groups were tested with the Wilcoxon rank-sum test and depicted through bar plots and heatmaps.

Immune cell fractions within each bulk sample were inferred through CIBERSORT-based deconvolution (Newman et al., 2015). These inferred fractions were then examined in three ways: contrasting their levels across risk strata, testing their correlation with the risk score itself, and, going a step further, cross-referencing the 20 highest-weighted model genes against each immune cell fraction to generate a bubble-plot summary of gene–cell relationships.

As a way to gauge whether risk stratification might translate into treatment implications, we applied the pRRophetic algorithm to predict drug-response scores for a panel of compounds from each TCGA-PAAD sample’s transcriptomic profile (Geeleher et al., 2014). The pRRophetic predictions were generated on the GDSC2016 natural-log-transformed IC50 phenotype scale and are therefore reported as predicted drug-response scores (GDSC ln [IC50] scale), rather than as raw IC50 concentrations. These scores were compared between the high- and low-risk strata. Representative drugs with significant and directionally clear differences were selected for visualization, including compounds with higher or lower predicted drug-response scores in the high-risk group. These results were considered computational predictions based on transcriptomic profiles and were used to suggest potential differences in therapeutic response.

### 2.9 Experimental validation of the key model gene KRT19

Because KRT19 showed the highest variable importance in the optimal RSF model, it was selected for functional validation. BxPC-3 cells were used as a representative human PDAC cell model for KRT19 overexpression and subsequent proliferation, migration, and invasion assays. Cells were maintained in complete culture medium supplemented with 10% fetal bovine serum and 1% penicillin-streptomycin at 37 °C in a humidified atmosphere containing 5% CO_2_. KRT19 overexpression plasmid and the corresponding negative-control vector were transfected into BxPC-3 cells using a conventional plasmid transfection procedure according to the manufacturer’s instructions to establish the OE-KRT19 and OE-NC groups, respectively. KRT19 overexpression was subsequently confirmed by Western blotting before functional assays were performed.

Successful overexpression at the protein level was confirmed via Western blotting. Total protein was extracted from transfected BxPC-3 cells using RIPA lysis buffer, with concentration determined by the BCA method. After resolving equal protein loads by SDS-PAGE, proteins were electrotransferred onto PVDF membranes, which were subsequently blocked in 5% non-fat milk before overnight incubation with primary antibodies against KRT19 and Tubulin. Following incubation with appropriate secondary antibodies, signals were captured using ECL detection reagent, with Tubulin serving as the loading control against which KRT19 protein changes were normalized.

Proliferative capacity was gauged through two complementary readouts. For the CCK-8 assay, transfected cells were seeded into 96-well plates, and cell proliferation was evaluated at 0, 24, 48, and 72 h. CCK-8 reagent was added at each time point according to the manufacturer’s instructions, and absorbance was measured at 450 nm. In parallel, a colony formation assay was conducted by sparsely plating transfected cells in 6-well dishes and allowing colonies to develop over time, after which they were fixed, stained with crystal violet, and manually counted.

Migratory behavior was captured through a scratch-wound assay: once transfected cells reached near-confluent monolayers in 6-well plates, a single scratch was drawn across the monolayer with the tip of a 200 μL pipette, after which loosened cells were washed away using PBS and the growth medium was swapped for one containing little or no serum. Wound images were acquired at 0 and 24 h under identical microscopic conditions. Cell migration was quantified according to changes in wound gap distance between the two time points, using the same measurement criteria for the OE-KRT19 and OE-NC groups.

To corroborate these migratory findings and additionally probe invasive potential, Transwell chambers were employed. In the migration setup, serum-starved transfected cells were placed in the upper compartment while serum-containing medium in the lower compartment served as the chemotactic stimulus. After incubation, cells remaining on the upper membrane surface were carefully removed, whereas cells that had migrated through the membrane were fixed and stained with crystal violet. Representative microscopic fields were photographed under identical magnification and imaging conditions, and migrated cells were quantified using consistent criteria across groups. For the invasion assay, the upper chamber membrane was precoated with Matrigel, and the remaining procedures were performed using the same protocol. The invasion setup followed an identical workflow except that the membrane was first coated with Matrigel. Each functional assay was independently repeated no fewer than three times.

### 2.10 Single-center clinical cohort and machine-learning-based prognostic modeling

An independent single-center cohort comprising 468 patients with PDAC was included to evaluate the prognostic value of routinely available clinicopathological characteristics. Overall survival (OS) was used as the primary outcome. Candidate predictors included demographic characteristics, tumor-related pathological features, serum tumor markers, and routine laboratory indicators. This study was approved by the Ethics Committee of Qingdao Central Hospital. Given the retrospective nature of the data collection, the requirement for informed consent was waived by the Ethics Committee.

Eight survival prediction approaches were evaluated, including CoxBoost, stepwise Cox regression, LASSO, RSF, elastic net, ridge regression, gradient boosting machine, and XGBoost. To assess model robustness, the cohort was repeatedly divided into training and testing sets at a ratio of 7:3 for 100 iterations. Each model was fitted in the training subset and evaluated in the corresponding testing subset using the C-index. Models were ranked according to their overall C-index distributions across repeated data splits, and the three top-performing models were retained for subsequent variable importance analysis.

Variable importance values generated by the three selected models were normalized to a range of 0–1. A weighted importance score was then calculated for each clinical variable according to its normalized importance and the mean C-index of the corresponding model. The number of top-performing models in which each variable was selected was also recorded to reflect the consistency of variable selection.

High-weight clinical variables were subsequently evaluated using univariate and multivariate Cox regression analyses. Hazard ratios, 95% confidence intervals, and P values were calculated to assess their associations with OS. A clinical nomogram integrating the selected variables was then developed to estimate individualized 1-, 3-, and 5-year survival probabilities. The discriminatory performance of the nomogram was evaluated using time-dependent ROC analysis.

### 2.11 Statistical analysis

All statistical analyses were performed using R software (version 4.4.2). Continuous variables were compared using the Wilcoxon rank-sum test for two groups and the Kruskal–Wallis test for multiple groups. Categorical variables were compared using the chi-square test or Fisher’s exact test, as appropriate. Correlations were evaluated using Spearman correlation analysis. Survival differences were assessed using the Kaplan-Meier method and log-rank test, while Cox regression analyses were used to estimate hazard ratios and 95% confidence intervals. Model discrimination was evaluated using the C-index and time-dependent ROC analysis. For the single-center cohort, C-index distributions were summarized across 100 repeated 7:3 training-testing splits. Experimental data are presented as the mean ± standard deviation and were compared using Student’s t-test. Multiple testing correction was performed using the Benjamini–Hochberg method when applicable. All tests were two-sided, and P < 0.05 was considered statistically significant.

## 3 Results

**FIGURE 1:**
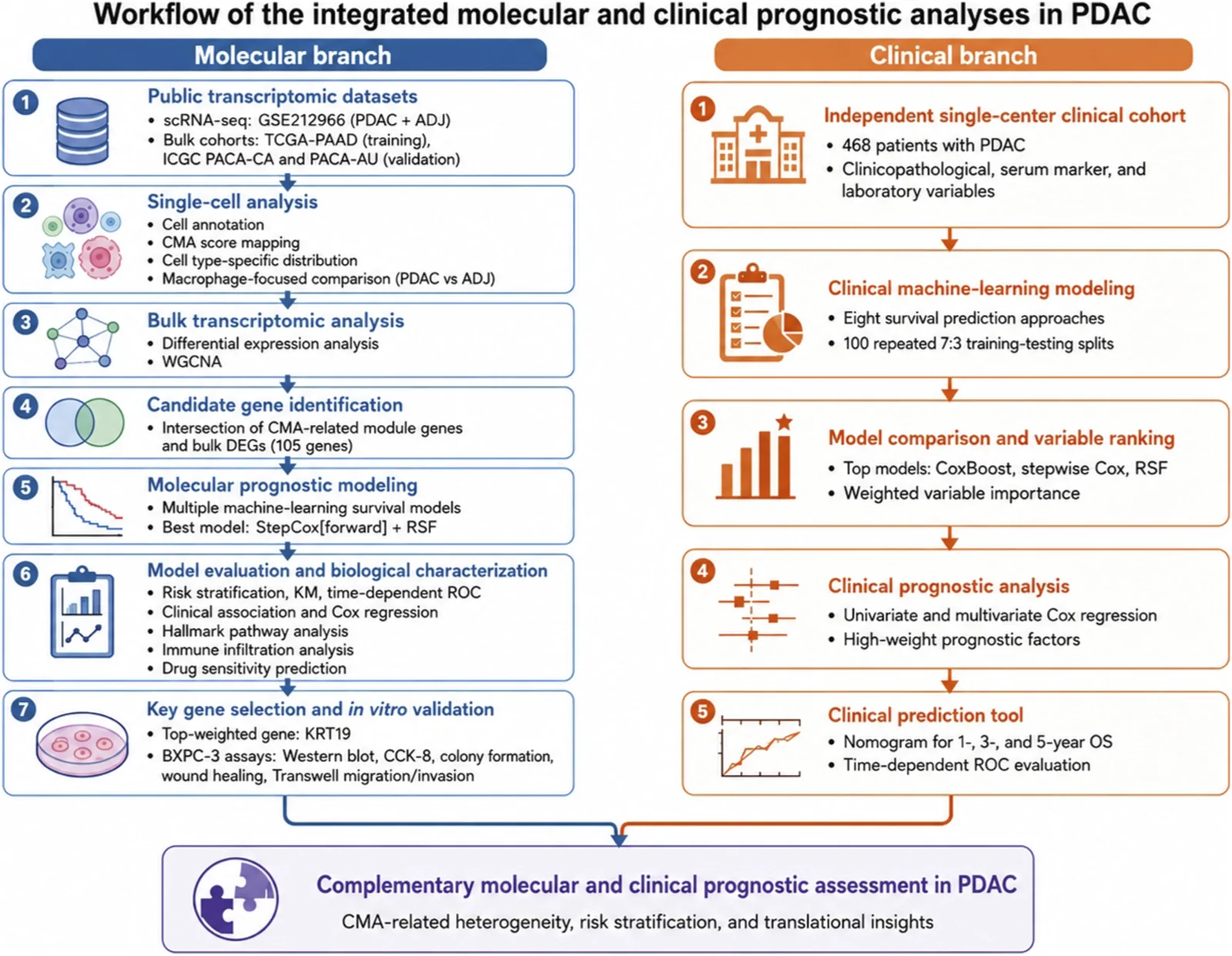
Workflow of the integrated molecular and clinical prognostic analyses in PDAC. Public transcriptomic datasets were used to characterize CMA-related states, construct and validate a molecular prognostic model, and perform KRT19 functional validation. An independent single-center cohort was analyzed to identify clinical prognostic factors and develop a clinical nomogram.

The overall workflow of the present study is shown in Figure 1.

### 3.1 Single-cell atlas revealed marked cellular specificity of CMA-related transcriptional activity in PDAC

**FIGURE 2:**
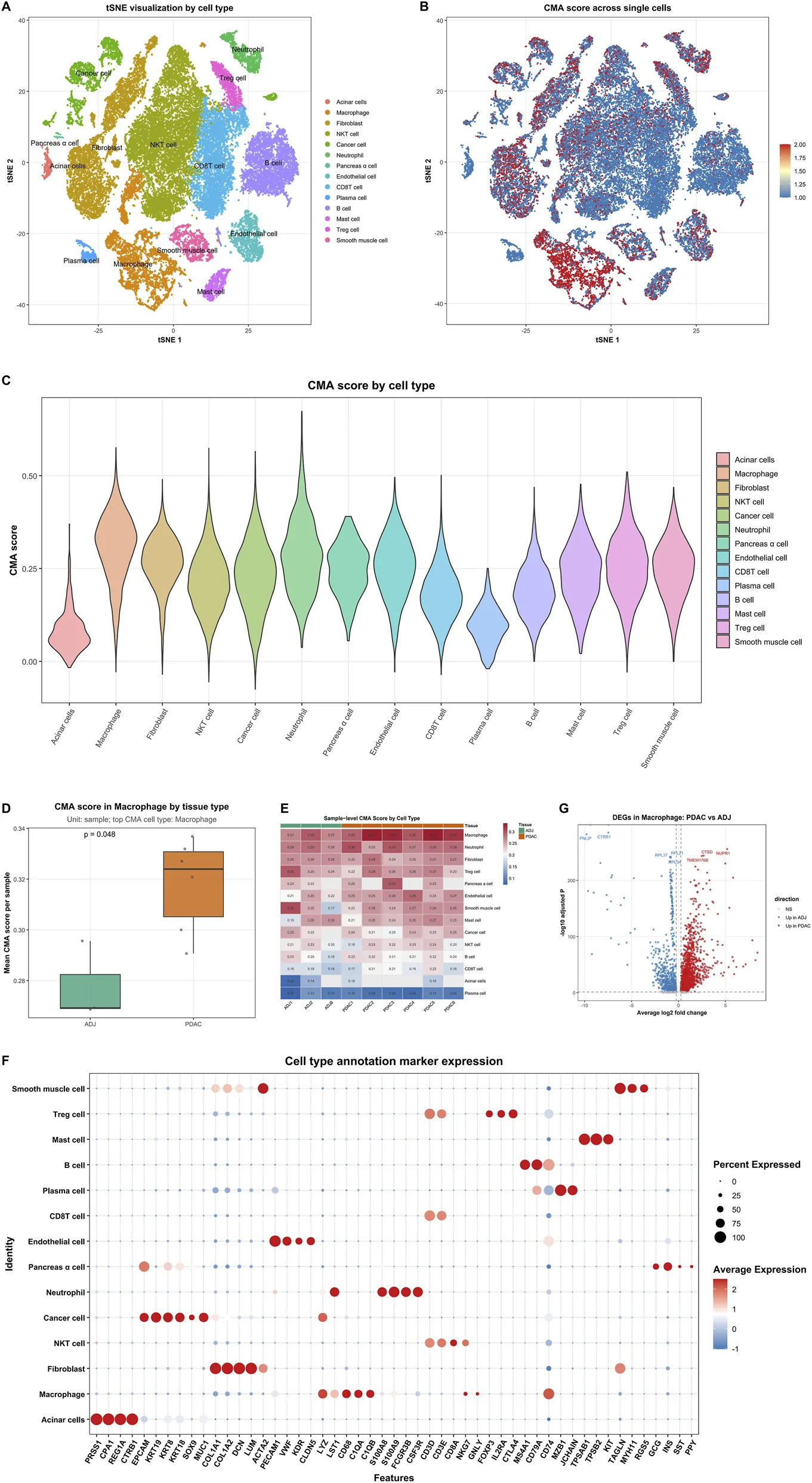
Single-cell characterization of CMA score distribution in PDAC. **(A)** tSNE plot depicting the cell type annotations derived from the GSE212966 dataset. **(B)** Projection of per-cell CMA scores onto the tSNE embedding. **(C)** Violin plots contrasting CMA score levels across the annotated cell populations. **(D)** Mean CMA score within the macrophage compartment, contrasted between adjacent normal (ADJ) and tumor (PDAC) tissue. **(E)** Sample-resolved heatmap depicting how CMA scores vary across cell types. **(F)** DotPlot illustrating marker gene expression profiles underlying the cell type calls. **(G)** Volcano plot highlighting genes differentially expressed between PDAC-derived and ADJ-derived macrophages. Differences in macrophage CMA scores between PDAC and ADJ samples were assessed using the Wilcoxon rank-sum test.

To characterize the cellular distribution of CMA-related transcriptional activity in the PDAC tissue ecosystem, we first analyzed the GSE212966 scRNA-seq dataset and visualized the major cell populations using tSNE. The cells were annotated as Acinar cells, macrophages, Fibroblast, NKT cell, Cancer cell, Neutrophil, Pancreas α cell, Endothelial cell, CD8T cell, Plasma cell, B cell, Mast cell, Treg cell, and Smooth muscle cell (Figure 2A). These cell populations covered the major tumor epithelial, immune, and stromal components of PDAC tissues, providing a basis for subsequent analysis of the cellular origins of CMA scores.

We then calculated CMA scores at the single-cell level and projected them onto the tSNE map. CMA scores showed clear heterogeneity across different cell populations rather than a uniform distribution across all cells (Figure 2B). Overall, relatively higher CMA scores were observed in several immune and stromal cell regions, suggesting that the CMA-related transcriptional state in PDAC was not restricted to Cancer cells but may also arise from multiple non-malignant components of the tumor microenvironment. Further comparison of CMA scores among cell types using violin plots showed marked differences across cell populations (Figure 2C). Macrophages, Neutrophil, Fibroblast, Endothelial cell, Treg cell, and Smooth muscle cell showed relatively higher CMA scores, whereas Acinar cells and Plasma cell showed relatively lower CMA scores. These results indicate that CMA-related transcriptional activity exhibits marked cellular specificity in PDAC single-cell atlas and may be particularly associated with myeloid and stromal cell states.

Given that macrophages showed a prominent CMA score distribution, we further compared sample-level mean macrophage CMA scores between ADJ and PDAC tissues. PDAC samples showed higher mean macrophage CMA scores than ADJ samples, and the difference reached statistical significance (P = 0.048, Figure 2D). This result shows that macrophages in PDAC tissues may display enhanced CMA-related transcriptional programs compared with those in adjacent tissues. The sample-level CMA score heatmap further showed that CMA scores varied across both sample origins and cell types. Several cell populations in PDAC samples displayed relatively higher CMA scores, with the change in macrophages being particularly evident (Figure 2E). These findings support that alterations in CMA-related states in PDAC are not driven by a single cell or sample but show a consistent pattern within the tumor tissue ecosystem.

As a cross-check on the accuracy of these assigned cell identities, we next inspected how well-established marker genes were expressed across the annotated populations. DotPlot analysis showed that Acinar cells highly expressed pancreatic exocrine markers such as PRSS1, CPA1, REG1A, and CTRB1; Cancer cells highly expressed epithelial and tumor-related markers such as EPCAM, KRT19, KRT8, KRT18, S100P, and MUC1; macrophages highly expressed myeloid markers including LYZ, CD68, CD14, C1QA, and C1QB; Fibroblast highly expressed COL1A1, COL1A2, DCN, and LUM; Endothelial cell highly expressed PECAM1, VWF, KDR, and CLDN5; NKT cell mainly expressed T/NK-related markers such as CD3D, CD3E, NKG7, and GNLY; and Treg cell expressed CD3D, CD3E, FOXP3, IL2RA, and CTLA4 (Figure 2F). These marker gene expression patterns were consistent with the corresponding cell type identities, supporting the biological reliability of the cell annotation.

Finally, we compared DEGs between PDAC and ADJ within macrophages. The volcano plot showed that NUPR1, CTSD, and TMEM176B were markedly upregulated in PDAC-derived macrophages compared with ADJ-derived macrophages, whereas pancreatic exocrine-related genes such as PNLIP and CTRB1 were downregulated (Figure 2G). These DEGs indicate clear transcriptional differences between PDAC-derived and ADJ-derived macrophages. Overall, Figure 2 shows that CMA scores exhibit marked cell type specificity in the PDAC single-cell atlas, with macrophages representing a prominent CMA-related cell population and PDAC-derived macrophages showing relatively increased CMA scores.

### 3.2 Narrowing down CMA-associated candidates through TCGA-based differential expression and co-expression network analysis

**FIGURE 3:**
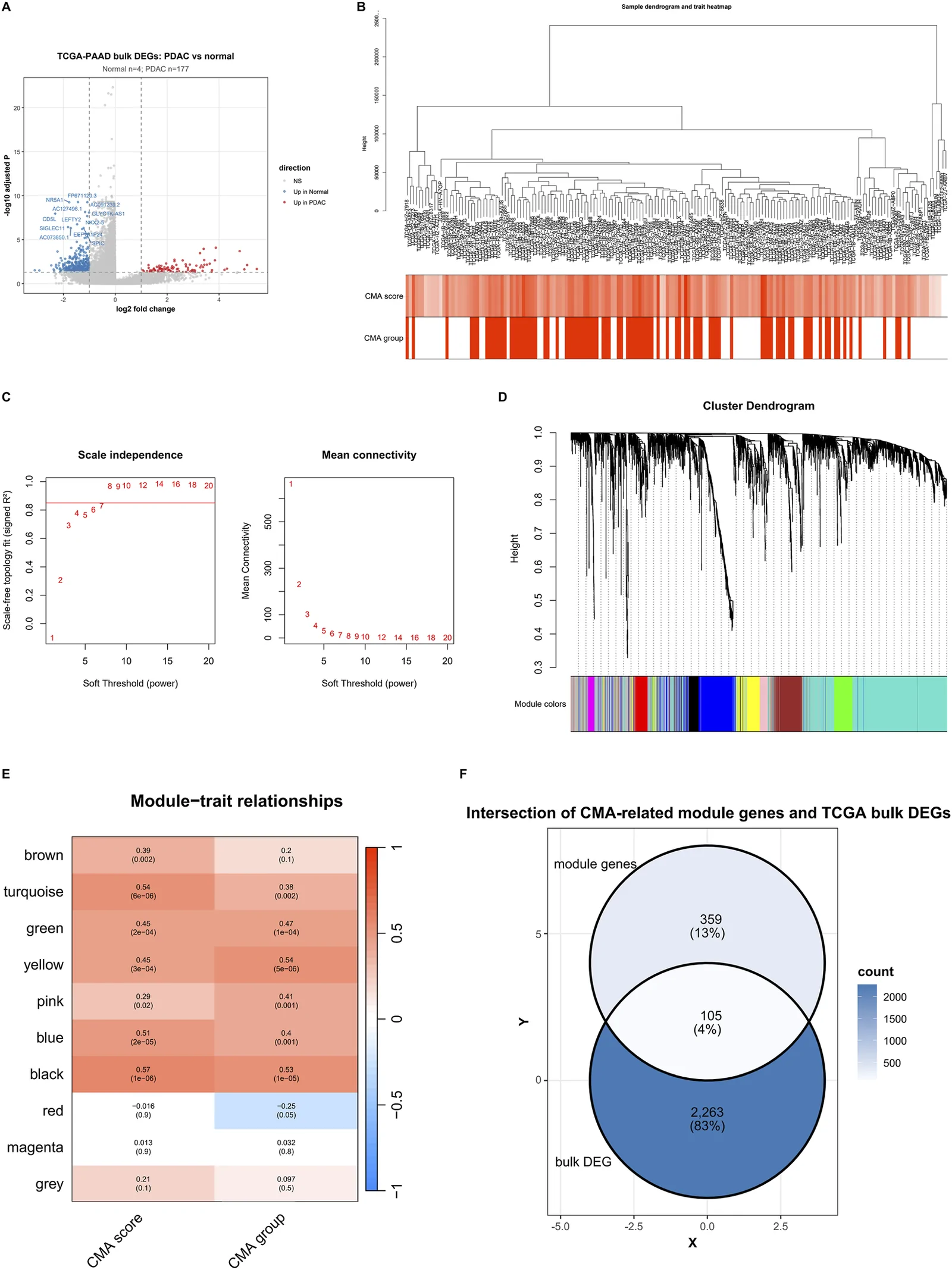
Identification of CMA-related candidate genes by TCGA bulk transcriptomic analysis and WGCNA. **(A)** Volcano plot contrasting gene expression between PDAC tumor and normal specimens in TCGA-PAAD. **(B)** Dendrogram of sample clustering paired with a trait heatmap reflecting CMA score and CMA group assignment. **(C)** Evaluation of the scale-free topology criterion and average connectivity across candidate soft-thresholding powers. **(D)** Dendrogram depicting how genes were partitioned into co-expression modules. **(E)** Heatmap summarizing the strength of association between each module and CMA-related traits. **(F)** Venn diagram depicting the shared gene set obtained by cross-referencing the CMA-associated module with TCGA-identified DEGs.

To support candidate gene screening at the bulk transcriptomic level, we compared gene expression between 177 PDAC tumor samples and 4 normal samples in the TCGA-PAAD cohort. The resulting differentially expressed genes were used as an initial filtering set for subsequent integration with the CMA-associated WGCNA module rather than as standalone evidence of tumor-specific expression alterations (Figure 3A).

We then performed WGCNA using the bulk CMA score as a continuous trait. The sample dendrogram combined with the CMA score and CMA group heatmap showed expression similarity among samples and their corresponding CMA status distribution (Figure 3B). During soft-thresholding power selection, the scale-free topology fit index reached a relatively high level while mean connectivity remained acceptable when the power was set to 8. Therefore, power = 8 was selected for co-expression network construction (Figure 3C). Based on this parameter, WGCNA identified multiple co-expression modules, and the cluster dendrogram showed gene clustering across different modules (Figure 3D).

Module-trait relationship analysis showed that several modules were positively correlated with CMA score and CMA group (Figure 3E). Among them, the black module showed the strongest correlation with CMA score (r = 0.57, P < 0.0001) and was also significantly positively correlated with CMA group (r = 0.53, P < 0.0001). In addition, the turquoise, blue, green, and yellow modules also showed positive correlations with CMA-related traits to varying degrees. Based on the strongest correlation with continuous CMA score, the black module was selected as the main CMA-related module for subsequent candidate gene screening.

To identify genes associated with both CMA status and the PDAC tumor state, we intersected the selected CMA-related module genes with TCGA bulk DEGs. The Venn diagram showed that the CMA-related module contained 464 genes, TCGA bulk DEGs contained 2,368 genes, and their intersection included 105 genes (Figure 3F). Among these 105 overlapping genes, 6 were upregulated in PDAC (logFC >1), whereas 99 were downregulated in PDAC (logFC < −1) relative to normal pancreatic tissues. This shared set of 105 genes was taken forward as the pool of CMA-linked candidates from which the machine-learning-based prognostic signature would subsequently be built and externally tested.

### 3.3 Building and cross-cohort testing of a CMA-linked risk model via comparative machine-learning screening

**FIGURE 4:**
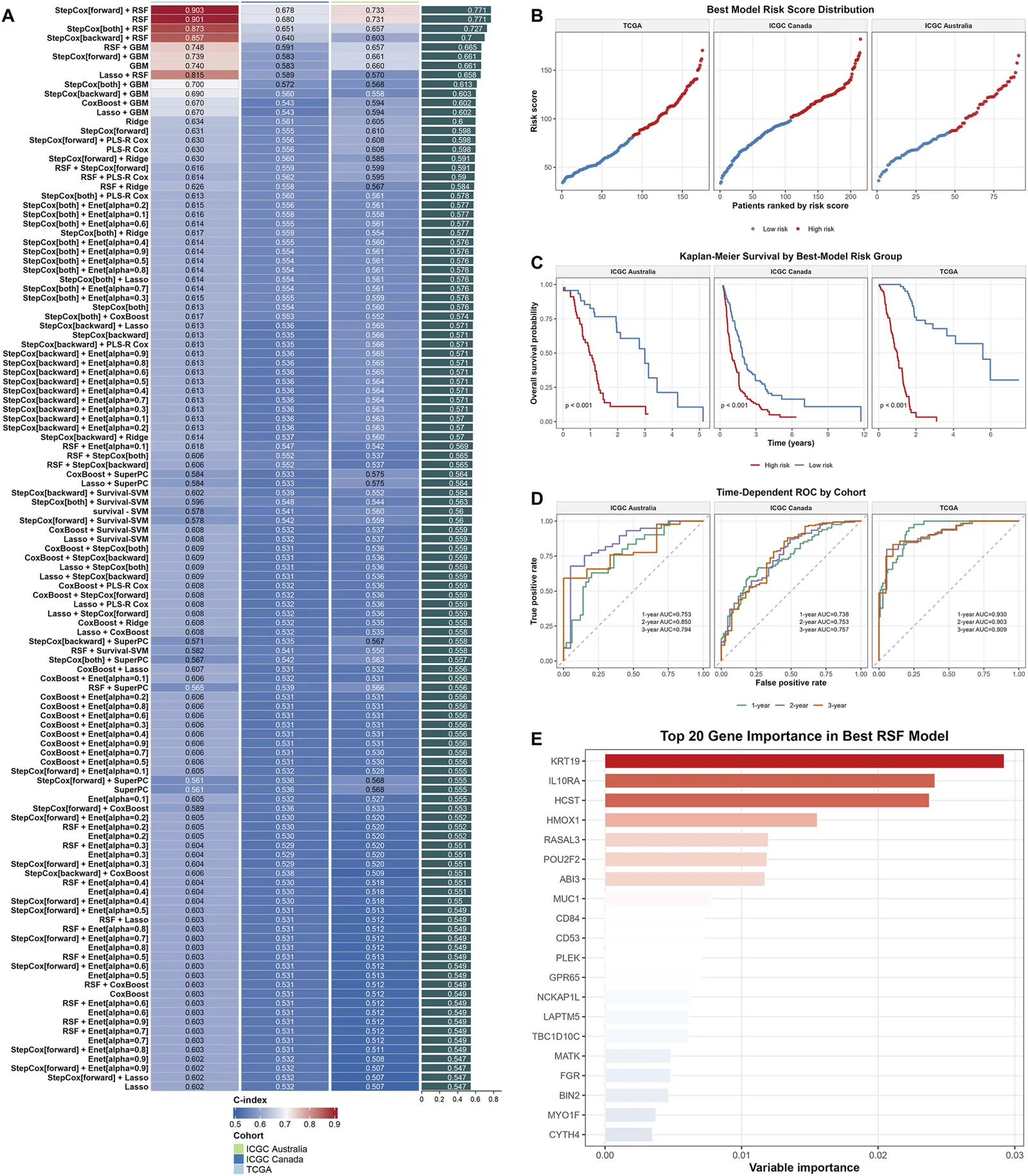
Building and validating the CMA-linked risk model across cohorts. **(A)** Heatmap of C-index values summarizing how each candidate algorithm performed within TCGA, ICGC Canada, and ICGC Australia. **(B)** Ranked risk score profiles for patients in each of the three cohorts, generated using the top-performing model. **(C)** Kaplan-Meier curves contrasting survival outcomes between risk strata across the three patient sets. **(D)** Time-dependent ROC curves capturing 1-, 2-, and 3-year predictive accuracy of the model in each cohort. **(E)** The twenty genes contributing most to variable importance within the selected RSF model.

Starting from the 105 candidate genes narrowed down in the preceding step, we proceeded to train a series of survival-oriented machine-learning models on the TCGA cohort, subsequently testing their generalizability against the two independent ICGC cohorts from Canada and Australia. Model selection proceeded by systematically benchmarking a wide array of survival-oriented algorithms—used both individually and in paired combinations—against their discriminative capacity as measured by the C-index. StepCox [forward] + RSF showed favorable overall performance, with a mean C-index of 0.771 and cohort-specific values of 0.903 in the TCGA dataset, 0.678 in the ICGC Canada cohort, and 0.733 in the ICGC Australia cohort (Figure 4A). Standalone RSF showed comparable performance. StepCox [forward] + RSF was retained for subsequent analyses because it incorporates an explicit feature-selection step before RSF modeling.

Applying this winning model, we derived a risk score for every patient and then split each cohort at the median value, designating those above the threshold as high-risk and the remainder as low-risk. Risk score distribution plots showed that patients could be continuously ranked by risk score in the TCGA, ICGC Canada, and ICGC Australia cohorts, with high-risk patients mainly distributed on the higher risk score side (Figure 4B). This result suggests that the model retained prognostic discrimination in both external validation cohorts, although its performance was lower than that observed in the training cohort.

Consistent with this stratification, Kaplan-Meier curves revealed a clear survival disadvantage for high-risk patients across all three cohorts examined. Whether in the TCGA discovery set or either of the two ICGC validation sets, the high-risk survival trajectory fell noticeably below that of the low-risk group, and this separation was confirmed by log-rank testing in every case (P < 0.001 throughout, Figure 4C)—evidence that the model’s prognostic power held up consistently across independent patient populations.

For time-dependent ROC results, the 1-, 2-, and 3-year AUC values were 0.930, 0.903, and 0.909 in the TCGA cohort; 0.738, 0.753, and 0.757 in the ICGC Canada cohort; and 0.753, 0.850, and 0.794 in the ICGC Australia cohort, respectively (Figure 4D). Although the AUC values in the external validation cohorts were lower than those in the TCGA training cohort, the model still maintained favorable predictive performance overall.

Finally, we ranked the top 20 important genes according to variable importance in the optimal RSF model. KRT19 showed the highest importance in the model, followed by IL10RA, HCST, HMOX1, RASAL3, POU2F2, ABI3, and other genes (Figure 4E). These genes constituted the main feature basis of the CMA-related prognostic model and were used for subsequent clinical association and functional interpretation analyses.

### 3.4 Association of the CMA-related risk score with clinical features and core model gene expression patterns

**FIGURE 5:**
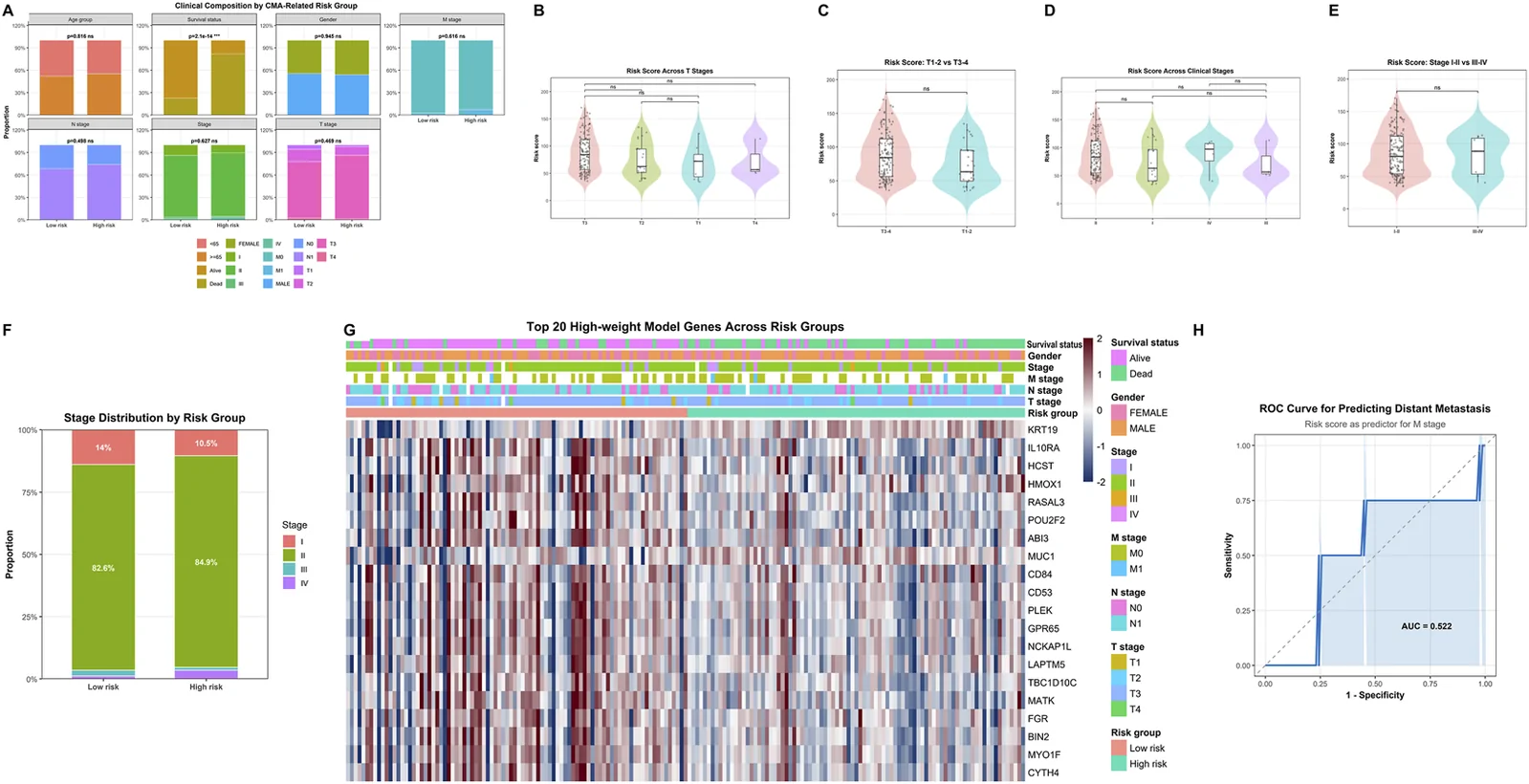
Clinical association and expression patterns of core model genes in the CMA-related risk groups. **(A)** Breakdown of clinical characteristics for patients assigned to two groups. **(B)** Distribution of risk scores stratified by individual T-stage categories, shown as violin plots. **(C)** Risk score contrast between the simplified T1–2 and T3–4 groupings. **(D)** Distribution of risk scores across individual clinical stage categories, shown as violin plots. **(E)** Comparison of risk scores between Stage I–II and Stage III–IV groups. **(F)** Stage distribution in the high-risk and low-risk groups. **(G)** Heatmap showing the expression patterns of the top 20 high weight model genes across risk groups and clinical annotations. **(H)** ROC curve evaluating the ability of the molecular risk score to discriminate patients with versus without distant metastasis.

Extending the clinical characterization introduced earlier, we examined whether the makeup of key clinicopathological variables differed depending on which risk stratum a patient fell into. Survival status stood out as the sole variable showing a pronounced imbalance, with deceased patients disproportionately concentrated in the high-risk stratum (Figure 5A). None of the remaining variables—age group, M stage, N stage, overall clinical stage, or T stage—showed a comparably skewed distribution across the two risk groups (Figure 5A). These results suggest that the risk score was closely associated with patient survival status but was not simply equivalent to traditional clinical staging variables.

We then compared the distribution of risk scores across different T stages and clinical stages. The risk score showed no obvious overall difference across T stages, and no significant difference was observed after combining patients into T1–2 and T3–4 groups (Figures 5B,C). In the clinical stage analysis, the overall difference in risk score among different stages was limited. After further combining patients into Stage I–II and Stage III–IV groups, the risk score still did not differ significantly between the two groups (Figures 5D,E). Stage distribution by risk group also showed that both groups were dominated by Stage II patients, with similar overall stage composition (Figure 5F).

To examine the expression characteristics of core model genes across risk groups, we displayed the expression heatmap of the top 20 high-weight genes in the optimal RSF model. These model genes showed clear expression heterogeneity among patients and were displayed together with risk group, survival status, and selected clinical variables (Figure 5G). KRT19 ranked highest in the preceding variable importance analysis and was therefore selected as the key gene for subsequent experimental validation.

Finally, we evaluated whether the molecular risk score could distinguish patients according to distant metastasis status. The ROC analysis yielded an AUC of 0.522 (Figure 5H), indicating that the molecular risk score did not meaningfully discriminate patients with versus without distant metastasis. This finding suggests that the prognostic information captured by the risk score is distinct from its ability to classify M stage.

### 3.5 Independent prognostic contribution of the CMA risk score and its link to Hallmark pathway activity

**FIGURE 6:**
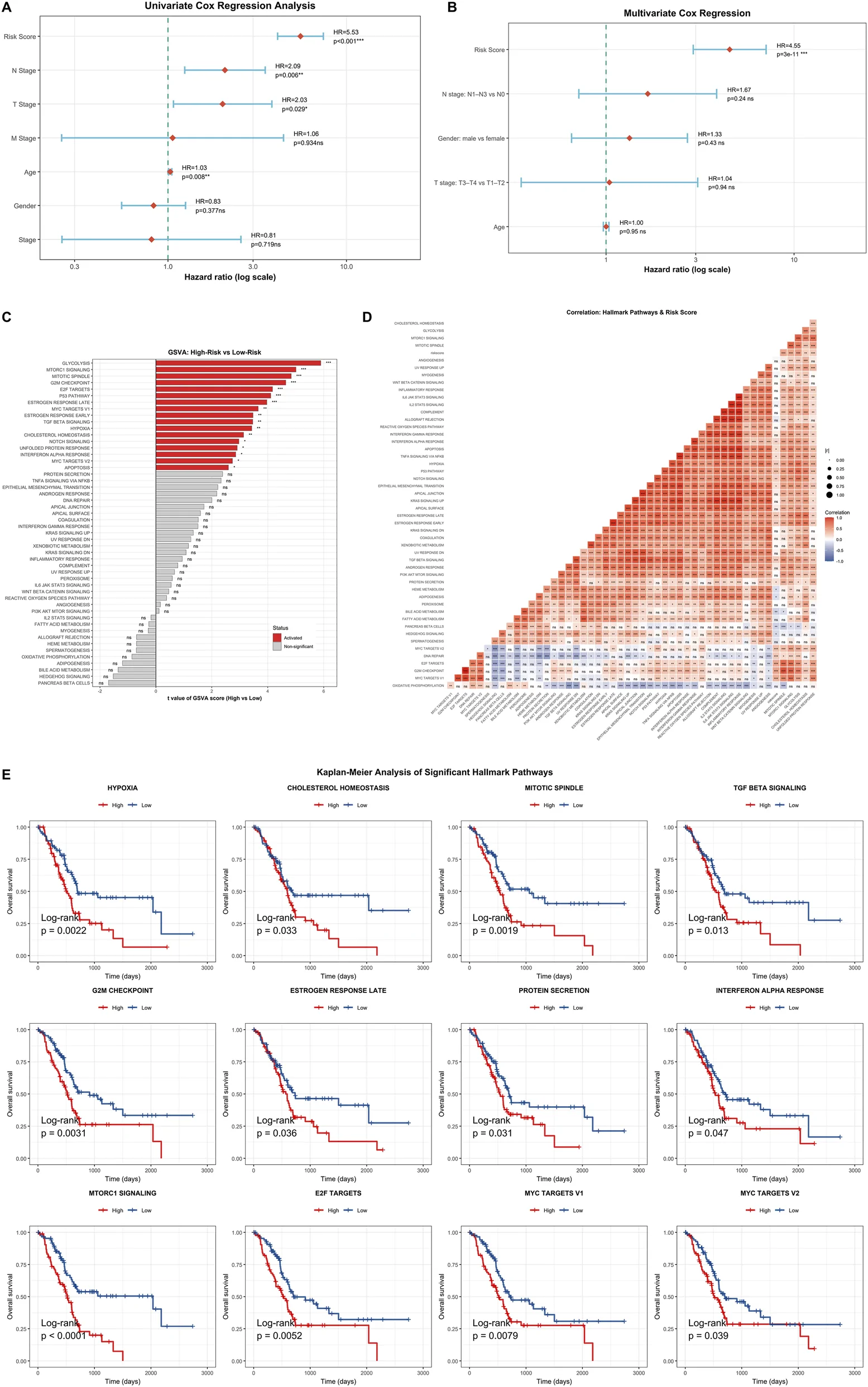
Prognostic independence of the risk score and its relationship to Hallmark pathway biology. **(A)** Forest plot from univariate Cox regression relating the risk score and clinical covariates to overall survival. **(B)** Forest plot from multivariate Cox regression testing whether the risk score retains prognostic value after adjustment. **(C)** GSVA-derived comparison of Hallmark pathway activity between high- and low-risk patients. **(D)** Matrix depicting correlation strength between the risk score and individual Hallmark pathway scores. **(E)** Kaplan-Meier survival curves for 12 representative Hallmark pathways showing significant associations with overall survival. Differences in Hallmark pathway activity between risk groups were assessed using the Wilcoxon rank-sum test, correlations were evaluated using Spearman correlation analysis, and survival differences were assessed using the Kaplan-Meier method and log-rank test. ns, not significant; *P < 0.05; **P < 0.01; ***P < 0.001; ****P < 0.0001.

As an initial test of prognostic relevance, univariate Cox regression was applied, revealing a strong and statistically robust link between the risk score and overall survival (HR = 5.53, P < 0.001). Alongside the risk score, N stage, T stage, and age also emerged as significant prognostic contributors, while M stage, sex, and overall clinical stage failed to reach significance in this analysis (Figure 6A). When these variables were entered together into a multivariate model, the risk score continued to hold independent prognostic significance (HR = 4.55, P < 0.001). In this model, age, gender, T group, and N group were not statistically significant, suggesting that the CMA-related risk score could serve as a prognostic indicator independent of these clinical variables (Figure 6B).

We next analyzed Hallmark pathway differences. GSVA showed that multiple Hallmark pathways were significantly increased in the high-risk group, including GLYCOLYSIS, MTORC1 SIGNALING, MITOTIC SPINDLE, G2M CHECKPOINT, E2F TARGETS, and MYC TARGETS, which are related to proliferation, metabolism, and the cell cycle (Figure 6C). Most remaining Hallmark pathways did not show significant differences, indicating that the high-risk group mainly exhibited more active proliferative and metabolic functional states.

To further examine the continuous relationship between risk score and Hallmark pathways, we constructed a correlation matrix between Hallmark pathway scores and the risk score. Proliferation-, cell cycle-, and metabolism-related pathways generally showed positive correlations with the risk score, whereas some Hallmark pathways showed negative correlations (Figure 6D). This result suggests that the CMA-related risk score may reflect broad functional state changes in PDAC samples rather than alteration of a single pathway alone.

Kaplan-Meier survival analysis was further performed for representative Hallmark pathways associated with patient survival. Higher activity of HYPOXIA, CHOLESTEROL HOMEOSTASIS, MITOTIC SPINDLE, TGF BETA SIGNALING, G2M CHECKPOINT, ESTROGEN RESPONSE LATE, PROTEIN SECRETION, INTERFERON ALPHA RESPONSE, MTORC1 SIGNALING, E2F TARGETS, MYC TARGETS V1, and MYC TARGETS V2 was associated with poorer overall survival (Figure 6E). These findings further support the association between the molecular risk score and unfavorable functional states in PDAC.

### 3.6 CMA-related risk score was associated with immune microenvironmental features

**FIGURE 7:**
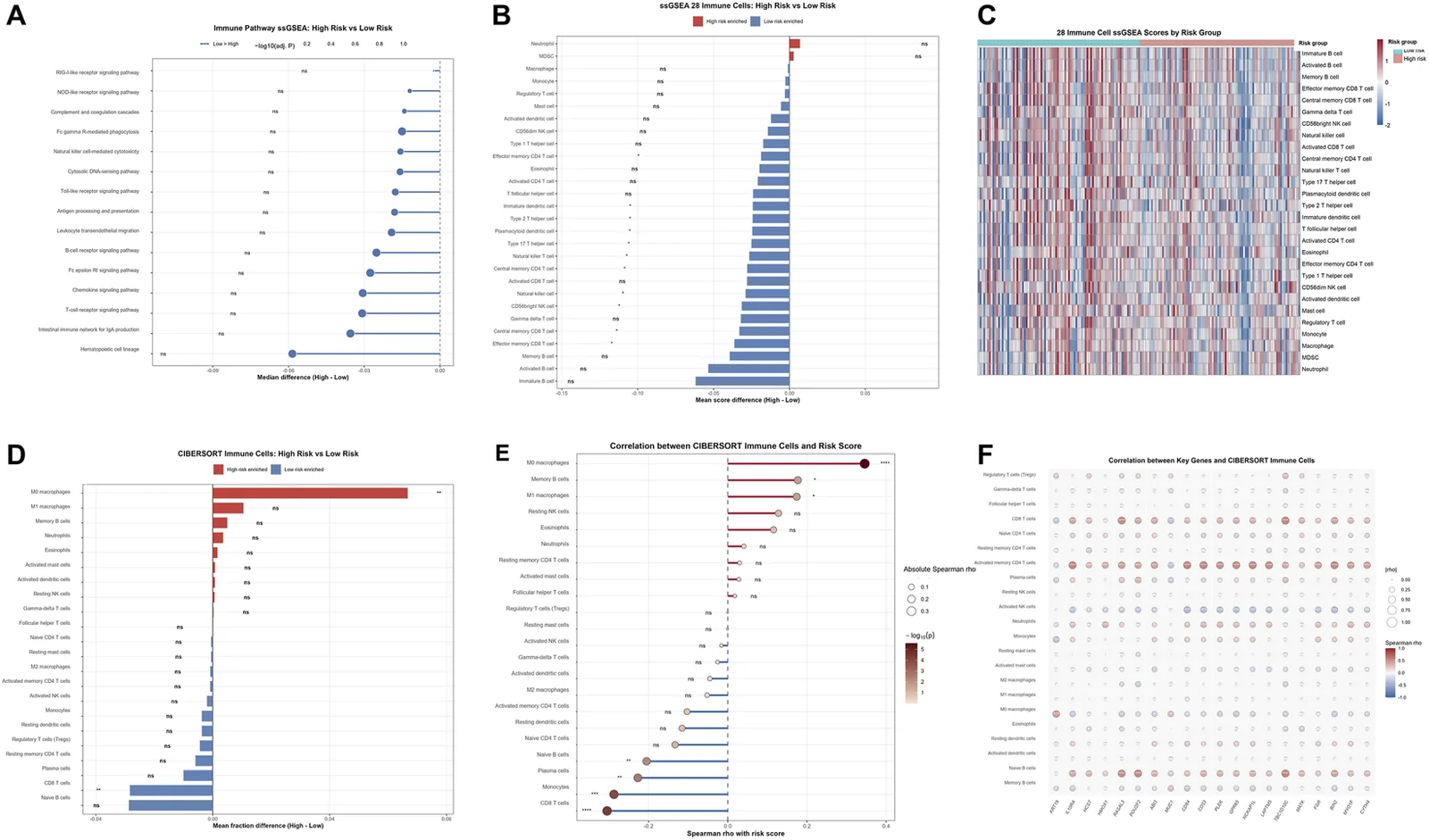
Immune landscape associated with the CMA-related risk score. **(A)** Comparison of immune pathway ssGSEA scores across risk strata. **(B)** Comparison of ssGSEA-derived scores for 28 immune cell subsets across risk strata. **(C)** Heatmap depicting the 28-subset ssGSEA profile for each patient, grouped by risk category. **(D)** Differences in CIBERSORT estimated immune cell fractions between the two groups. **(E)** Correlations between CIBERSORT immune cell fractions and risk score. **(F)** Bubble plot mapping correlations between core model genes and CIBERSORT-derived immune cell fractions. Differences in immune scores or estimated immune cell fractions between risk groups were assessed using the Wilcoxon rank-sum test. Correlations between risk score, model genes, and immune cell fractions were evaluated using Spearman correlation analysis. ns, not significant; *P < 0.05; **P < 0.01; ***P < 0.001; ****P < 0.0001.

Given that a number of genes contributing to the model carried recognized immune functions, we turned our attention to how the risk score related to the broader immune context of PDAC tumors. Starting with pathway-level immune activity, ssGSEA scores showed essentially no separation between high- and low-risk patients across any of the immune-related pathways examined, indicating that this particular readout of immune activity was largely insensitive to risk stratification (Figure 7A).

Shifting to a finer-grained cellular view, we compared ssGSEA-derived scores spanning 28 distinct immune cell subsets between the two risk strata. With the exception of neutrophils and MDSCs, which trended modestly higher among high-risk patients, the overall pattern favored the low-risk group, which displayed elevated scores for most subsets—including a range of T cell, NK cell, B cell, and dendritic cell populations (Figure 7B). Visualizing all 28 subset scores together as a heatmap reinforced this pattern, with the low-risk cohort visibly enriched for higher immune cell scores overall (Figure 7C).

Seeking corroboration from an orthogonal deconvolution approach, we turned to CIBERSORT-estimated cell fractions. This analysis pinpointed two immune populations with clear risk-group differences: M0 Macrophages were enriched among high-risk patients, while CD8^+^ T cells were more abundant in the low-risk group (Figure 7D); the remaining cell types showed no comparable degree of separation. Consistent with the group-wise comparison, correlation testing linked the risk score positively to M0 Macrophages abundance, while revealing inverse relationships with CD8^+^ T cells, Monocytes, Plasma cells, and naive B cells (Figure 7E)—together pointing to a pattern whereby escalating risk scores track with expanding M0 macrophage presence alongside a contraction of several other immune compartments.

As a final step linking gene-level and cell-level data, we mapped how the twenty highest-weighted model genes correlated with each CIBERSORT-derived cell fraction. The resulting bubble plot revealed that KRT19, MUC1, and several other immune-related genes within the model each showed distinct, gene-specific correlation patterns across different immune populations (Figure 7F), underscoring that the CMA-based signature is intertwined with shifts in the PDAC immune cell landscape.

### 3.7 CMA-related risk groups suggested potential differences in drug sensitivity

**FIGURE 8:**
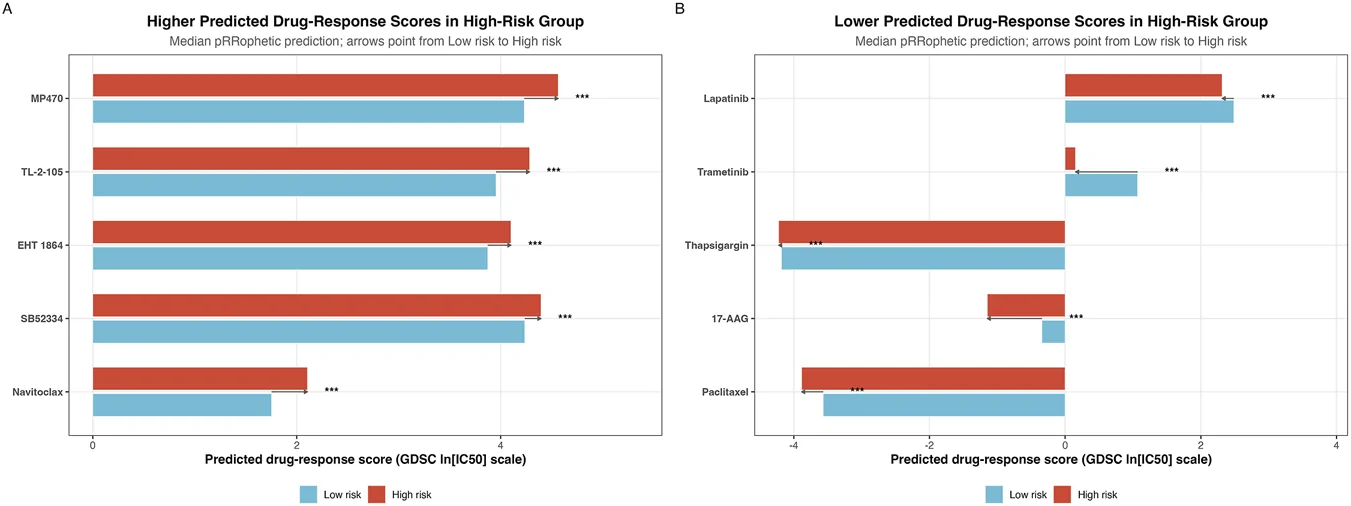
Predicted drug-response differences between CMA-related risk groups. **(A)** Compounds for which the high-risk group showed higher predicted drug-response scores relative to the low-risk group. **(B)** Compounds with lower predicted drug-response scores in the high-risk group. Bars represent the median pRRophetic-predicted drug-response score on the GDSC ln (IC50) scale, and arrows indicate the direction of change from the low-risk to the high-risk group. Differences between the high- and low-risk groups were assessed using the Wilcoxon rank-sum test. ns, not significant; *P < 0.05; **P < 0.01; ***P < 0.001; ****P < 0.0001.

Several compounds showed higher predicted drug-response scores in the high-risk group, including MP470, TL-2–105, EHT 1864, SB52334, and navitoclax (Figure 8A), indicating relatively lower predicted sensitivity to these compounds. In contrast, lapatinib, trametinib, thapsigargin, 17-AAG, and paclitaxel showed lower predicted drug-response scores in the high-risk group (Figure 8B), indicating relatively greater predicted sensitivity. Among them, trametinib, 17-AAG, and paclitaxel showed particularly pronounced decreases in predicted drug-response scores. Overall, these findings suggest distinct predicted drug-response patterns between the two molecular risk groups.

### 3.8 KRT19 overexpression promoted PDAC cell proliferation, migration, and invasion

**FIGURE 9:**
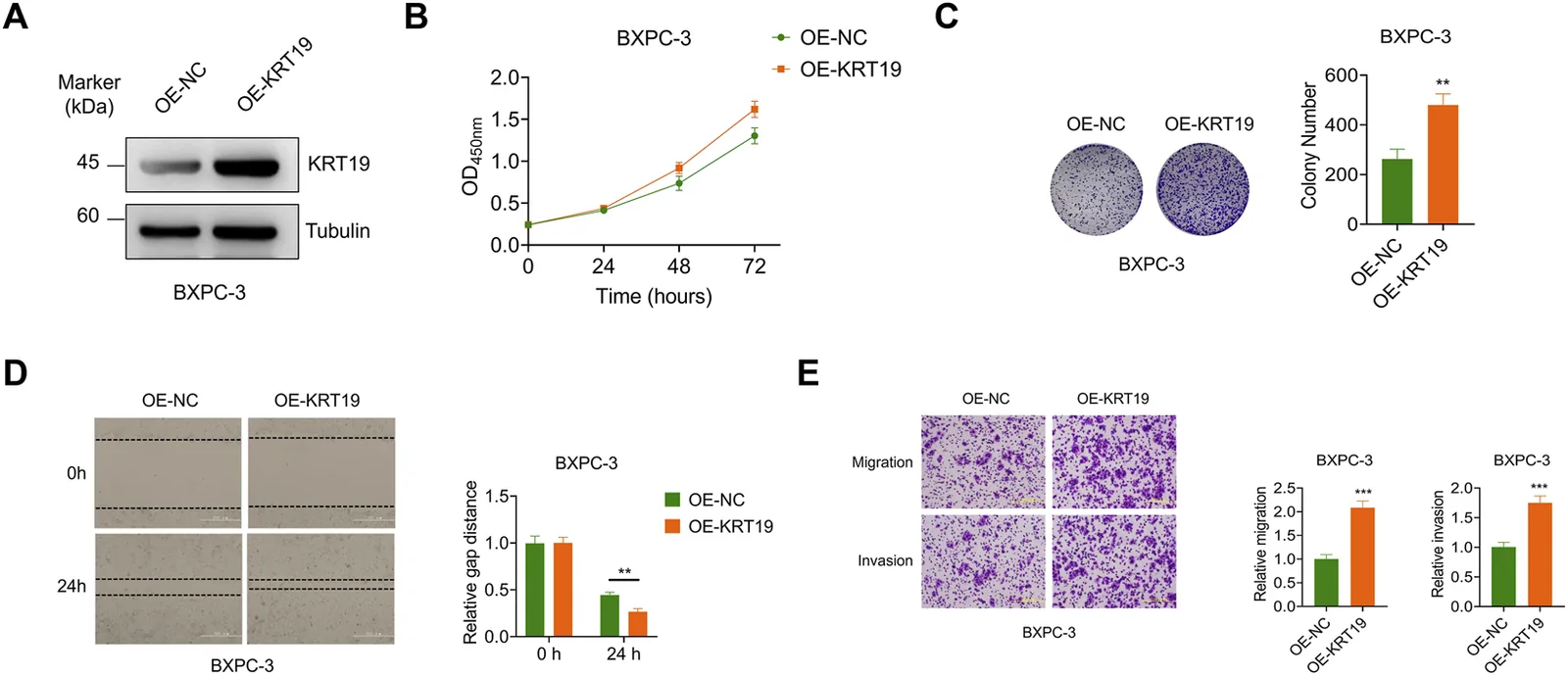
KRT19 overexpression promotes proliferation, migration, and invasion of PDAC cells. **(A)** Western blot verification of elevated KRT19 protein levels in BxPC-3 cells following transfection. **(B)** CCK-8 assay showing the effect of KRT19 overexpression on BxPC-3 cell proliferation. **(C)** Colony formation assay showing increased colony formation after KRT19 overexpression. **(D)** Wound healing assay showing enhanced migratory ability in KRT19-overexpressing BxPC-3 cells. **(E)** Transwell-based assessment of migration and invasion in KRT19-overexpressing versus control cells. Experimental data are presented as the mean ± standard deviation from at least three independent experiments. Comparisons between the OE-KRT19 and OE-NC groups were performed using Student’s t-test. ns, not significant; *P < 0.05; **P < 0.01; ***P < 0.001; **P < 0.0001.

Based on the preceding machine-learning model analysis, KRT19 ranked highest in variable importance in the optimal RSF model and was selected for experimental validation. A KRT19 overexpression model was first established in BxPC-3 cells, and KRT19 protein expression was assessed by Western blot. Western blot results confirmed that the overexpression strategy had worked as intended: KRT19 protein levels were substantially elevated in the OE-KRT19 group relative to OE-NC controls (Figure 9A).

Turning to proliferative capacity, we assessed this through both CCK-8 and colony formation readouts. Cells overexpressing KRT19 grew at a faster rate than OE-NC controls according to the CCK-8 data (Figure 9B), and this was echoed by the colony formation assay, in which the OE-KRT19 group produced a greater number of colonies (Figure 9C)—together indicating that elevated KRT19 levels boost the proliferative output of PDAC cells.

Migratory capacity was next examined via wound healing assay. By the 24 h mark, the gap had closed more extensively in KRT19-overexpressing cells than in controls, a difference that statistical comparison confirmed as significant (Figure 9D)—indicating that raising KRT19 expression facilitates PDAC cell migration.

Rounding out the functional characterization, Transwell assays were used to directly compare migratory and invasive behavior. Relative to OE-NC cells, substantially more OE-KRT19 cells traversed the membrane in both the migration and invasion setups, pointing to a clear gain in both motility and invasive potential following KRT19 overexpression (Figure 9E). Collectively, these experiments converge on a consistent picture: elevating KRT19 expression drives BxPC-3 cells toward a more proliferative, migratory, and invasive phenotype, lending functional weight to its designation as a top-ranked feature within the CMA-related risk model.

### 3.9 Machine-learning analysis identifies clinically accessible prognostic factors in an independent single-center cohort

**FIGURE 10:**
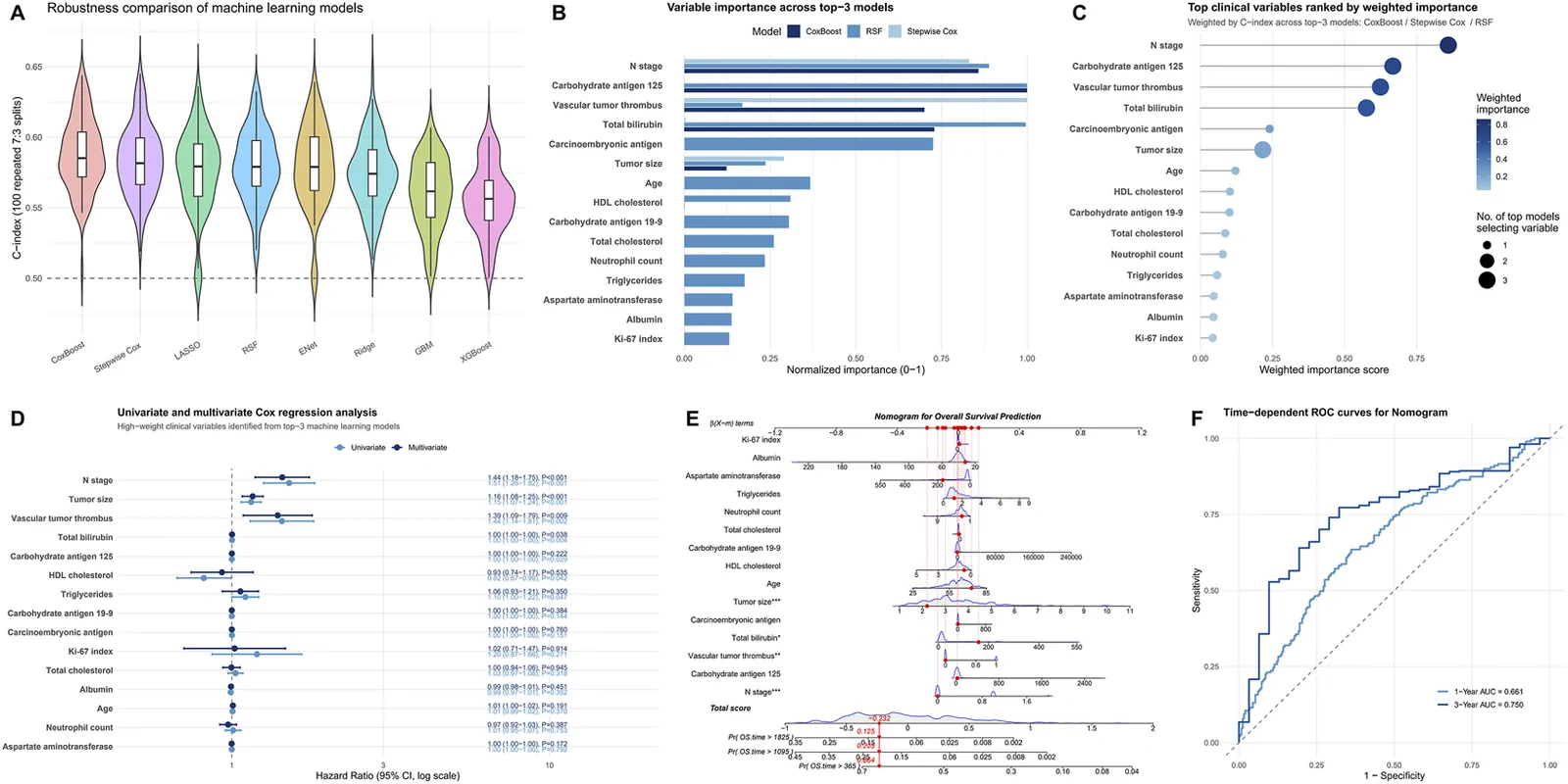
Machine-learning-based clinical prognostic modeling in an independent single-center PDAC cohort. **(A)** Robustness comparison of eight survival prediction models based on C-index distributions across 100 repeated 7:3 training-testing splits. **(B)** Normalized importance of clinical variables across the three top-performing models, including CoxBoost, RSF, and stepwise Cox regression. **(C)** Ranking of clinical variables according to weighted importance scores across the three selected models. Point color represents the weighted importance score, whereas point size indicates the number of top-performing models in which the variable was selected. **(D)** Univariate and multivariate Cox regression analyses of high-weight clinical variables associated with OS. **(E)** Clinical nomogram integrating the selected variables to estimate 1-, 3-, and 5-year survival probabilities. **(F)** Time-dependent ROC curves evaluating the 1- and 3-year predictive performance of the clinical nomogram.

The independent single-center cohort included 468 patients with PDAC, and their baseline clinicopathological characteristics are summarized in Table 1. To complement the transcriptome-derived molecular model with a clinically accessible prognostic framework, eight survival prediction approaches were evaluated using routinely collected clinicopathological and laboratory variables. Model robustness was assessed using 100 repeated 7:3 training-testing splits. CoxBoost, stepwise Cox regression, and RSF showed relatively favorable overall C-index distributions and were therefore selected for subsequent variable importance analysis (Figure 10A).

**TABLE 1:** Baseline clinicopathological characteristics of the single-center PDAC cohort.

Characteristic	Available n	Overall cohort (n = 468)
Demographic and clinical characteristics		
Age, years	468	63.5 (57–69)
Gender, n (%)	468	​
Male	​	288 (61.5)
Female	​	180 (38.5)
Body mass index, kg/m^2^	456	23.4 (21.2–25.33)
Diabetes, yes, n (%)	468	119 (25.4)
Alcohol history, yes, n (%)	467	110 (23.6)
Smoking history, yes, n (%)	467	151 (32.3)
Neoadjuvant chemoradiotherapy, yes, n (%)	468	63 (13.5)
Laboratory and serum markers		
Neutrophil count, ×10^9^/L	467	3.4 (2.8–4.33)
Lymphocyte count, ×10^9^/L	467	1.45 (1.18–1.84)
Albumin, g/L	468	40.3 (37.4–42.7)
Total bilirubin, μmol/L	468	18.34 (11.77–118.72)
Alanine aminotransferase, U/L	468	27.35 (14.23–166.57)
Aspartate aminotransferase, U/L	468	23.95 (16–102.1)
Triglycerides, mmol/L	459	1.24 (0.85–1.98)
Total cholesterol, mmol/L	459	4.88 (4.03–5.74)
High-density lipoprotein cholesterol, mmol/L	448	1.11 (0.8–1.39)
Low-density lipoprotein cholesterol, mmol/L	454	2.96 (2.34–3.62)
Blood glucose, mmol/L	466	6.04 (5.19–7.73)
Carcinoembryonic antigen, ng/mL	463	3.65 (2.21–6.89)
Carbohydrate antigen 19–9, U/mL	464	173.8 (46.59–628.59)
Carbohydrate antigen 125, U/mL	435	15.32 (10.38–28.56)
Tumor characteristics		
Tumor location, n (%)	466	​
Pancreatic head	​	243 (52.1)
Pancreatic body	​	150 (32.2)
Pancreatic tail	​	73 (15.7)
Tumor size, cm	463	3.2 (2.55–4.25)
Ki-67 index, %	461	20 (5–40)
Vascular tumor thrombus, yes, n (%)	464	119 (25.6)
Perineural invasion, yes, n (%)	463	309 (66.7)
T stage, n (%)	466	​
T0	​	1 (0.2)
T1	​	48 (10.3)
T2	​	239 (51.3)
T3	​	101 (21.7)
T4	​	77 (16.5)
N stage, n (%)	463	​
N0	​	304 (65.7)
N1	​	143 (30.9)
N2	​	16 (3.5)
Differentiation grade, n (%)	465	​
Well differentiated	​	5 (1.1)
Moderately differentiated	​	246 (52.9)
Poorly differentiated	​	214 (46.0)
Double-duct sign, yes, n (%)	467	160 (34.3)
Survival outcomes		
Death events, n (%)	468	337 (72.0)
Observed survival time, days	468	385 (241.8–602.2)

Data are presented as median (interquartile range) or n (%). Percentages were calculated using the available observations for each variable. Zero values for CA125, HDL cholesterol, CEA, triglycerides, total cholesterol, LDL cholesterol, CA19-9, and tumor size were treated as missing in accordance with the preprocessing procedure.

Variable importance differed across the three selected models (Figure 10B). N stage showed high importance in all three models, whereas carbohydrate antigen 125 (CA125), vascular tumor thrombus, total bilirubin (TB), carcinoembryonic antigen (CEA), and tumor size also made prominent contributions in at least one model. After integrating the normalized importance values according to model-specific C-index weights, N stage ranked first, followed by CA125, vascular tumor thrombus, and TB (Figure 10C). CEA and tumor size showed intermediate weighted importance, whereas age, high-density lipoprotein cholesterol, carbohydrate antigen 19–9 (CA19-9), total cholesterol, neutrophil count, triglycerides, aspartate aminotransferase, albumin, and Ki-67 index contributed relatively less to the integrated ranking.

The high-weight variables were further evaluated using Cox regression analysis. Univariate analysis identified significant associations of N stage, tumor size, vascular tumor thrombus, TB, CA125, high-density lipoprotein cholesterol, and triglycerides with OS. After adjustment for the other clinical variables, N stage, tumor size, vascular tumor thrombus, and TB remained independently associated with survival (Figure 10D). These findings indicate that lymph node involvement, tumor burden, vascular invasion, and bilirubin level capture complementary aspects of clinical prognostic heterogeneity in PDAC.

A clinical nomogram incorporating the selected clinicopathological and laboratory variables was subsequently established to estimate individualized 1-, 3-, and 5-year survival probabilities (Figure 10E). Time-dependent ROC analysis showed AUC values of 0.661 and 0.750 for predicting 1- and 3-year OS, respectively (Figure 10F). Thus, the model demonstrated moderate prognostic discrimination based solely on routinely available clinical information, providing a clinically accessible perspective complementary to the transcriptome-derived CMA-related risk model.

## 4 Discussion

The present study investigated PDAC prognostic heterogeneity from complementary molecular and clinical perspectives. Single-cell analysis revealed cell type-specific variation in CMA-related transcriptional states, with macrophages showing prominent CMA scores and PDAC-derived macrophages exhibiting higher scores than their ADJ-derived counterparts. By integrating TCGA differential expression analysis with WGCNA, 105 CMA-related candidate genes were identified, and the StepCox [forward] + RSF model retained prognostic discrimination in the two independent ICGC cohorts, although its performance was lower than that observed in TCGA. The molecular risk score was associated with proliferative and metabolic pathways, immune infiltration, and predicted drug sensitivity, while KRT19 overexpression promoted malignant phenotypes in PDAC cells. Importantly, the newly included single-center cohort provided an independent clinical perspective, identifying routinely available prognostic factors and supporting the construction of a clinical nomogram. Thus, the molecular and clinical models captured distinct but complementary dimensions of PDAC prognosis.

CMA contributes to selective protein degradation and cellular adaptation to metabolic and environmental stress. This process may be particularly relevant in PDAC, which is characterized by hypoxia, nutrient deprivation, dense stroma, and extensive immune remodeling. In the present study, PDAC-derived macrophages showed increased CMA-related transcriptional activity and elevated expression of NUPR1, CTSD, and TMEM176B. Hamidi et al. reported that NUPR1 supports pancreatic tumor development by protecting malignant cells against stress-induced apoptosis (Hamidi et al., 2012). Ruiz-Blázquez et al. demonstrated that CTSD-dependent lysosomal activity contributes to macrophage functional remodeling (Ruiz-Blázquez et al., 2024), whereas Segovia et al. linked TMEM176B to myeloid immune regulation (Segov et al., 2019). Together with the role of tumor-associated macrophages in immunosuppression and therapeutic resistance described by Poh et al. (Poh and Ernst, 2021), these findings suggest that stress-response and lysosomal programs may participate in macrophage reprogramming within the PDAC microenvironment. However, the CMA score used here reflects a CMA-related transcriptional state rather than a direct measurement of functional CMA flux. Because the scoring gene set includes components involved in broader autophagy and lysosomal regulation, the observed differences should be interpreted as alterations in CMA-associated transcriptional programs rather than definitive evidence of increased LAMP2A-dependent CMA activity.

The molecular prognostic model was developed from genes associated with both CMA-related co-expression patterns and malignant tissue status, thereby reducing the contribution of unrelated transcriptomic signals. Its prognostic performance across independent cohorts suggests that the selected features retained a degree of cross-cohort stability. High-risk tumors showed enhanced proliferative, cell-cycle, and metabolic pathway activity. Chang et al. described glycolytic reprogramming as an important contributor to pancreatic cancer growth and metastasis (Chang et al., 2022), while Driscoll et al. demonstrated that mTOR signaling promotes PDAC development (Driscoll et al., 2016). The high-risk group also exhibited increased M0 macrophages and reduced CD8^+^ T cells, consistent with the favorable prognostic role of tumor-associated cytotoxic T cells reported by Carstens et al. (2017) and the adverse immune pattern associated with M0 macrophages described by Xu et al. (2023). Predicted sensitivity to trametinib, 17-AAG, and paclitaxel should be considered hypothesis-generating rather than evidence of clinical response, although previous studies by Awasthi et al. (2018), Zhang et al. (2010), and Von Hoff et al. (2013) provide a biological and therapeutic basis for further evaluation. KRT19 showed the highest importance in the molecular model. Consistent with the findings of Zhou et al. (2023) and Shi et al. (2024), KRT19 overexpression enhanced PDAC cell proliferation, colony formation, migration, and invasion, supporting its functional relevance to aggressive tumor behavior. The specific relationship between KRT19 and CMA-related regulation warrants further investigation.

A major extension of the present study was the independent analysis of routinely collected clinical information from the single-center cohort. CoxBoost, stepwise Cox regression, and RSF showed favorable robustness across repeated data partitions. Clinical variables reflecting nodal involvement, tumor burden, vascular invasion, and systemic status contributed substantially to prognostic prediction. These findings are clinically plausible because nodal involvement, tumor size, and vascular invasion reflect tumor dissemination and invasive burden. Liu et al. found that serum CA125 was closely associated with metastatic burden in pancreatic cancer (Liu et al., 2016), and Napoli et al. subsequently identified CA125 as an independent prognostic marker in resected pancreatic cancer (Napoli et al., 2023). Li et al. also reported that tumor size and suspicious lymph nodes were associated with unfavorable survival after PDAC resection (Li et al., 2022). The clinical nomogram achieved AUCs of 0.661 and 0.750 for 1- and 3-year OS, respectively, indicating moderate discrimination using readily available variables. Similar to the present approach, Zhuang et al. incorporated pathological stage, biochemical indicators, and tumor markers into a practical PDAC prognostic nomogram (Zhuang et al., 2021). These clinicopathological indicators of tumor dissemination and invasive burden are conceptually consistent with the proliferative, metabolic, and invasive features observed in the molecular high-risk group and with the malignant phenotypes promoted by KRT19 overexpression. Because the single-center cohort lacked paired transcriptomic data, the clinical analysis should not be regarded as direct validation of the CMA-related signature. Instead, it provides a clinically accessible prognostic framework that complements the biological information captured by the molecular model.

Several limitations should be noted. First, the molecular model was developed from retrospective public datasets and requires prospective multicenter validation. In addition, the TCGA-PAAD cohort contained only four normal samples, which limited the robustness of the tumor-versus-normal differential expression analysis; therefore, these DEGs were used only as an initial filtering layer in combination with WGCNA. Second, the single-center cohort lacked paired transcriptomic data, so the relationship between the molecular and clinical models could only be inferred. Nevertheless, because the two models capture complementary biological and clinicopathological information, their integration may further improve prognostic performance, including the AUC, which should be verified in future matched cohorts. Third, the CMA score did not directly measure functional CMA activity, and KRT19 validation was limited to overexpression in a single cell line. In summary, this study identified CMA-related cellular heterogeneity, established an externally validated molecular prognostic model, confirmed the functional relevance of KRT19, and developed an independent clinical prediction model. These complementary findings provide a broader framework for prognostic stratification in PDAC.

## 5 Conclusion

This study characterized CMA-related cellular heterogeneity in PDAC and established a molecular prognostic model that retained prognostic discrimination in two independent validation cohorts. KRT19 was identified as a key model gene and promoted malignant phenotypes in PDAC cells. In parallel, the single-center clinical model and nomogram provided a complementary approach for clinically accessible prognostic assessment.

## Data Availability

The raw data supporting the conclusions of this article will be made available by the authors, without undue reservation.
